# Supplementary material for: Explaining rising caesarean section rates in urban Nepal: A mixed-methods study
Source: PLoS One. 2025 Feb 26;20(2):e0318489. doi: 10.1371/journal.pone.0318489 (PMC11864527; doi:10.1371/journal.pone.0318489)
Supplement: S1 Table — (PDF) [file pone.0318489.s001.pdf]

PLOS

Lorem Ipsum

Lorem ipsum dolor sit amet, consectetur adipiscing elit. Mauris maximus fringilla ligula, in malesuada erat tempor ac. Quisque dapibus posuere turpis, vel aliquam massa vehicula non.

S1 Table: Sociodemographic Characteristics of Women

Sociodemographic Characteristics	Category	Frequency	Percentage
Age of mother	<20	51	7.7
	20-24	224	33.9
	25-29	206	31.2
	30-34	143	21.6
	35+	37	5.6
Gestational Age	<30	10	1.5
	30-36	89	13.5
	37-40	540	81.7
	>40	22	3.3
Parity	Nulliparous	380	57.5
	Multiparous	281	42.5
Number of Pregnancy	Single	657	99.4
	Multiple	4	0.6
Foetal lie	Longitudinal	660	99.8
	Transverse	1	0.2
Foetal Presentation	Cephalic	630	95.3
	Breech	30	4.5
	Other	1	0.2
Labour induction	No	590	89.3
	Yes	71	10.7
Previous Delivery Type	Normal	185	65.8
	CS	96	34.2
Current Delivery type	Normal	329	49.8
	CS	332	50.2
Birth other	1	380	57.5
	2	200	30.3
	3+	81	12.2
Medical Condition	Yes	29	4.4
	No	632	95.6
BOH	Yes	53	8.0
	No	608	92.0
Number of ANC Visit	0	6	0.9
	1-3	233	35.2
	4+	422	63.8
Religion	Hindu	471	71.3
	Buddhist	158	23.9
	Christian	25	3.8
	Muslim	7	1.1
Ethnicity	High	258	39.0
	Middle	376	56.9
	Dalit	27	4.1
Place of Residence	Urban	345	52.2
	Rural	316	47.8
