# Supplementary material for: Explaining rising caesarean section rates in urban Nepal: A mixed-methods study
Source: PLoS One. 2025 Feb 26;20(2):e0318489. doi: 10.1371/journal.pone.0318489 (PMC11864527; doi:10.1371/journal.pone.0318489)
Supplement: S3 Table — (PDF) [file pone.0318489.s003.pdf]

PLOS

Lorem Ipsum

Lorem ipsum dolor sit amet, consectetur adipiscing elit. Mauris maximus fringilla ligula, in malesuada erat tempor ac. Quisque dapibus posuere turpis, vel aliquam massa vehicula non.

S3 Table: Indications of CS in elective and emergency CS

Indications of CS	Elective No. (%)	Emergency No. (%)	Total No. (%)
• Medical indications			
Foetal distress	-	48 (14.5)	48 (14.5)
Previous CS	91 (27.4)	-	91 (27.4)
Cephalopelvic disproportion (CPD)	16 (5.1)	18 (5.7)	36 (10.8)
Breech	24 (7.2)	2 (0.6)	26 (7.8)
Oligohydramnios	11 (3.3)	4 (1.2)	15 (4.5)
Premature rupture of membrane (PROM)	1 (0.3)	11 (3.3)	12 (3.6)
Meconium-stained liquor	-	9 (2.7)	9 (2.7)
Non-progression of labour (NPOL)	-	8 (1.8)	8 (2.4)
HDP (Hypertensive disorders in pregnancy)	5 (1.8)	2 (0.6)	7 (2.1)
Non-reactive cardiotocography (NRCTG)	-	7 (2.1)	7 (2.1)
Prolong labour	-	5 (1.5)	5 (1.5)
Cord round neck	-	4 (1.2)	4 (1.2)
Antepartum haemorrhage (APH)	-	4 (1.2)	4 (1.2)
Previous CS & breech	3 (0.9)	-	3 (0.9)
Deep transverse arrest (DTA)	-	3 (.9)	3 (0.9)
Invitro-fertilisation (IVF)/subfertility	3 (0.9)	-	3 (0.9)
Twin	2 (0.6)	-	2 (0.6)
Bad obstetric history (BOH)	2 (0.9)	-	2 (0.6)
Fail induction	-	2 (0.9)	2 (0.9)
Intra-uterine growth retardation (IUGR)	2 (0.9)	-	2 (0.6)
Rh negative	2 (0.6)	-	2 (0.6)
Maternal distress	-	2 (0.9)	2 (0.9)
Spinal bifida/intrauterine abnormality	1 (0.3)	1 (0.3)	2 (0.9)
Maternal medical condition (seizure, hypothyroidism)	1 (0.3)	1 (0.3)	2 (0.9)
Preterm	-	1 (0.3)	1 (0.3)
Previous CS & scar tenderness	-	1 (0.3)	1 (0.3)
Transverse lie	1 (0.3)	-	1 (0.3)
Previous CS & twin	1 (0.3)	-	1 (0.3)
Young primigravida	1 (0.3)	-	1 (0.3)
Breech & twin	1 (0.3)	-	1 (0.3)
Chorioamnionitis	-	1 (0.3)	1 (0.3)
• Non-medical indications			
Non specified reason2	6 (1.8)	13 (3.9)	19 (5.7)
Maternal request	9 (2.7)	-	9 (2.7)
Total	185 (55.7)	147 (44.2)	332 (100.0)

S3_table 3_202501225048-1.tif This is a preview of your figure rendered on a simulated PLOS journal page.

Maecenas ac est sit amet odio sollicitudin euismod. In risus odio, convallis a neque ac, varius ultricies arcu. Vestibulum et quam iaculis, ultricies odio et, molestie magna. Suspendisse vehicula purus id turpis eleifend, et convallis dui dignissim. Praesent tempus elit a metus sollicitudin, sed fringilla nulla porttitor. Nullam in tempus massa. Nunc maximus magna massa, nec volutpat risus rhoncus ut. Fusce quis ante sem. Aenean nulla nibh, tempus sit amet rhoncus at, eleifend vel risus. Sed dictum, sem ultrices elementum pharetra, lacus diam volutpat orci, scelerisque semper dui lacus ut enim.

Suspendisse in nunc id lacus commodo consequat. Proin semper aliquam varius. Fusce vitae neque aliquam nisi ultrices sodales vitae ut enim. Vivamus nec dictum ipsum. Sed condimentum ante eu urna tincidunt tincidunt. In ac lacus nec ipsum viverra volutpat posuere vel lacus. Class aptent taciti sociosqu ad litora torquent per conubia
