# Supplementary material for: Explaining rising caesarean section rates in urban Nepal: A mixed-methods study
Source: PLoS One. 2025 Feb 26;20(2):e0318489. doi: 10.1371/journal.pone.0318489 (PMC11864527; doi:10.1371/journal.pone.0318489)
Supplement: S4 Table — (PDF) [file pone.0318489.s004.pdf]

PLOS

Lorem Ipsum

Lorem ipsum dolor sit amet, consectetur adipiscing elit. Mauris maximus fringilla ligula, in malesuada erat tempor ac. Quisque dapibus posuere turpis, vel aliquam massa vehicula non.

S4 Table: Indications of CS in Nullipara and Multipara

Indications of CS	Nullipara No. (%)	Multipara No. (%)	Total No. (%)
• Medical indications			
Foetal distress	38 (11.5)	10 (3.0)	47 (14.4)
Previous CS	-	91 (27.4)	9 1 (27.4)
Cephalopelvic disproportion (CPD)	32 (9.6)	4 (1.8)	36 (10.8)
Breech	19 (5.7)	7 (2.1)	26 (7.8)
Oligohydramnios	12 (3.6)	7 (2.1)	15 (4.5)
Premature rupture of membrane (PROM)	9 (2.7)	3 (0.9)	12 (3.6)
Meconium-stained liquor	6 (1.8)	3 (0.9)	9 (2.7)
HDP (Hypertensive disorders in pregnancy)	7 (2.1)	1 (0.3)	8 (2.4)
Non-progression of labour (NPOL)	6 (1.8)	2 (0.6)	8 (2.4)
Non-reactive cardiotocography (NRCTG)	4 (1.2)	3 (0.9)	7 (2.1)
Prolong labour	3 (0.9)	2 (0.6)	5 (1.5)
Cord round neck	3 (0.9)	1 (0.3)	4 (1.2)
Antepartum haemorrhage (APH)	2 (0.6)	2 (0.6)	4 (1.2)
Previous CS & breech	-	3 (0.9)	3 (0.9)
Deep transverse arrest (DTA)	3 (0.6)	-	3 (0.9)
Twin	1 (0.3)	2 (06)	3 (0.9)
Invitro-fertilisation (IVF)/subfertility	3 (0.9)	-	3 (0.9)
Bad obstetric history (BOH)	2 (0.6)	-	2 (0.6)
Fail induction	-	2 (0.6)	2 (0.9)
Intra-uterine growth retardation (IUGR)	2 (0.6)	-	2 (0.9)
Rh negative	1 (0.3)	1 (0.3)	2 (0.9)
Maternal distress	2 (0.6)	-	2 (0.9)
Intrauterine foetal abnormality	2 (0.6)	-	2 (0.9)
Maternal medical condition (seizure, hypothyroidism)	1 (0.3)	1 (0.3)	2 (0.9)
Preterm	1 (0.3)	-	1 (0.3)
Previous CS & scar tenderness	-	1 (0.3)	1 (0.3)
Transverse lie	-	1 (0.3)	1 (0.3)
Previous CS & twin	-	1 (0.3)	1 (0.3)
Young primi	1 (0.3)	-	1 (0.3)
Breech & twin	1 (0.3)	-	1 (0.3)
Chorioamnionitis	1(0.3)	-	1 (0.3)
• Non-medical indications			
Non specified reason	13 (3.9)	6 (1.8)	19 (5.7)
Maternal request	6 (1.8)	3 (0.9)	9 (2.7)
Total	180 (57.2)	152 (45.8)	332 (100)

test-test-1 This is a preview of your figure rendered on a simulated PLOS journal page.

Maecenas ac est sit amet odio sollicitudin euismod. In risus odio, convallis a neque ac, varius ultricies arcu. Vestibulum et quam iaculis, ultricies odio et, molestie magna. Suspendisse vehicula purus id turpis eleifend, et convallis dui dignissim. Praesent tempus elit a metus sollicitudin, sed fringilla nulla porttitor. Nullam in tempus massa. Nunc maximus magna massa, nec volutpat risus rhoncus ut. Fusce quis ante sem. Aenean nulla nibh, tempus sit amet rhoncus at, eleifend vel risus. Sed dictum, sem ultrices elementum pharetra, lacus diam volutpat orci, scelerisque semper dui lacus ut enim.

Suspendisse in nunc id lacus commodo consequat. Proin semper aliquam varius. Fusce vitae neque aliquam nisi ultrices sodales vitae ut enim. Vivamus nec dictum ipsum. Sed condimentum ante eu urna tincidunt tincidunt. In ac lacus nec ipsum viverra volutpat posuere vel lacus. Class aptent taciti sociosqu ad litora torquent per conubia
