# Supplementary material for: Explaining rising caesarean section rates in urban Nepal: A mixed-methods study
Source: PLoS One. 2025 Feb 26;20(2):e0318489. doi: 10.1371/journal.pone.0318489 (PMC11864527; doi:10.1371/journal.pone.0318489)
Supplement: S5 Table — (PDF) [file pone.0318489.s005.pdf]

Lorem Ipsum

Lorem ipsum dolor sit amet, consectetur adipiscing elit. Mauris maximus fringilla ligula, in malesuada erat tempor ac. Quisque dapibus posuere turpis, vel aliquam massa vehicula non.

S5 Table: Robson ten group classification contribution to overall CS rate

Robson Group	Number women in each group	Total number vaginal births	Total number of CS	Group Size (%)	CS rate in each group (%)	Absolute group contribution to overall CS rate (%)	Relative group contribution to total CS rate (%)
Nulliparous, single cephalic, =>37 weeks in spontaneous labour	219	130	89	33.1	40.6	13.4	26.8
Nulliparous, single cephalic, =>37 weeks in induced labour or CS before labour	91	33	58	13.8	63.7	8.8	17.5
Multiparous, excluding previous CS, single cephalic, =>37 weeks in spontaneous labour	132	103	29	20	22.0	4.4	8.7
Multiparous, single cephalic, =>37 weeks in induced labour or prelabour CS	35	20	15	5.3	42.9	2.3	4.5
Previous CS, single cephalic, =>37 weeks) (13.9%)	92	0	92	13.9	100.0	13.9	27.7
All nulliparous breeches)	19	0	19	2.9	100.0	2.9	5.7
All multiparous breeches (including previous CS)	10	0	10	1.5	100.0	1.5	3.1
All multiple pregnancies (including previous CS)	4	0	4	0.6	100.0	0.6	1.2
All abnormal lies (including previous CS)	1	0	1	0.1	100.0	0.1	0.3
All single, cephalic, =<36 weeks (including previous CS)	58	43	15	8.8	25.9	2.3	4.5
Total	661	329	332	100.0	-	50.2	100.0

test-test-1 This is a preview of your figure rendered on a simulated PLOS journal page.

Maecenas ac est sit amet odio sollicitudin euismod. In risus odio, convallis a neque ac, varius ultricies arcu. Vestibulum et quam iaculis, ultricies odio et, molestie magna. Suspendisse vehicula purus id turpis eleifend, et convallis dui dignissim. Praesent tempus elit a metus sollicitudin, sed fringilla nulla porttitor. Nullam in tempus massa. Nunc maximus magna massa, nec volutpat risus rhoncus ut. Fusce quis ante sem. Aenean nulla nibh, tempus sit amet rhoncus at, eleifend vel risus. Sed dictum, sem ultrices elementum pharetra, lacus diam volutpat orci, scelerisque semper dui lacus ut enim.
