# Supplementary material for: Explaining rising caesarean section rates in urban Nepal: A mixed-methods study
Source: PLoS One. 2025 Feb 26;20(2):e0318489. doi: 10.1371/journal.pone.0318489 (PMC11864527; doi:10.1371/journal.pone.0318489)
Supplement: S6 Table — (PDF) [file pone.0318489.s006.pdf]

Lorem Ipsum

Lorem ipsum dolor sit amet, consectetur adipiscing elit. Mauris maximus fringilla ligula, in malesuada erat tempor ac. Quisque dapibus posuere turpis, vel aliquam massa vehicula non.

S6 Table: Contribution of Robson groups to overall CS rate in two hospitals

Robson Groups	PMWH						KMH						P value
	Total no. in each group	Total no. of CS	Group Size (%)	Group CS rate (%)	Absolute group contribution to overall CS rate (%)	Relative group contribution overall CS (%)	Total no. in each group	Total no. of CS	Group size (%)	Group CS rate (%)	Absolute group contribution to overall CS rate (%)	Relative group contribution to overall CS (%)	
1	146	46	37.9	31.5	11.9	32.2	73	43	26.4	58.9	15.6	22.7	0.001*
2	47	18	12.2	38.3	4.7	12.6	44	40	15.9	90.9	14.5	21.2	0.001*
3	81	13	21.0	16	3.4	9.1	51	16	18.5	31.4	5.8	8.5	0.052
4	17	6	4.4	35.3	1.5	4.2	18	9	6.5	50	3.3	4.8	0.500
5	29	29	7.5	100	7.5	20.3	63	63	22.8	100	22.8	33.3	-
6	13	13	3.4	100	3.4	9.1	6	6	2.2	100	2.2	3.2	-
7	6	6	1.6	100	1.6	4.2	4	4	1.5	100	1.4	2.1	-
8	3	3	0.8	100	0.8	2	1	1	0.4	100	0.4	0.5	-
9	1	1	0.3	100	0.2	0.7	0	0	0.0	0	0.0	0.0	-
10	42	8	10.9	19	2.1	5.6	16	7	5.8	43.7	2.5	3.7	0.091
Total	385	143	100.0		37.1	100	276	189	100		68.5	100	0.001*

test-test-1 This is a preview of your figure rendered on a simulated PLOS journal page.

Maecenas ac est sit amet odio sollicitudin euismod. In risus odio, convallis a neque ac, varius ultricies arcu. Vestibulum et quam iaculis, ultricies odio et, molestie magna. Suspendisse vehicula purus id turpis eleifend, et convallis dui dignissim. Praesent tempus elit a metus sollicitudin, sed fringilla nulla porttitor. Nullam in tempus massa. Nunc maximus magna massa, nec volutpat risus rhoncus ut. Fusce quis ante sem. Aenean nulla nibh, tempus sit amet rhoncus at, eleifend vel risus. Sed dictum, sem ultrices elementum pharetra, lacus diam volutpat orci, scelerisque semper dui lacus ut enim.

Suspendisse in nunc id lacus commodo consequat. Proin semper aliquam varius. Fusce vitae neque aliquam nisi ultrices sodales vitae ut enim. Vivamus nec dictum ipsum. Sed condimentum ante eu urna tincidunt tincidunt. In ac lacus nec ipsum viverra volutpat posuere vel lacus. Class aptent taciti sociosqu ad litora torquent per conubia nostra, per inceptos himenaeos. Morbi rhoncus ipsum quis lorem hendrerit, at vulputate massa tempus. Ut arcu nisl, gravida vitae risus ultricies, porta venenatis massa. Cras dignissim, enim at faucibus aliquam, sapien nisl eleifend dolor, vel mollis nulla nisi id ipsum. Pellentesque vehicula ultricies risus sit amet faucibus. Praesent sit amet mi ac est faucibus accumsan. Praesent pulvinar sit amet orci auctor feugiat.
