# Supplementary material for: Explaining rising caesarean section rates in urban Nepal: A mixed-methods study
Source: PLoS One. 2025 Feb 26;20(2):e0318489. doi: 10.1371/journal.pone.0318489 (PMC11864527; doi:10.1371/journal.pone.0318489)
Supplement: S7 Table — (PDF) [file pone.0318489.s007.pdf]

Lorem Ipsum

Lorem ipsum dolor sit amet, consectetur adipiscing elit. Mauris maximus fringilla ligula, in malesuada erat tempor ac. Quisque dapibus posuere turpis, vel aliquam massa vehicula non.

S7 Table: Robson ten group classification and risk ratio in study sample

Robson Group	Total number women in each group	Total number vaginal births	Total number CS	Size of each group (%)	CS rate in each group	CS Risk Ratio each group	95% CI	P-value
1.Nulliparous, single cephalic,≥37 weeks in spontaneous labour	219	130	89	33.1	40.6	0.74	0.62 - 0.89	0.0005*
2.Nulliparous, single cephalic, ≥37 weeks induced labour or CS before labour	91	33	58	13.8	63.7	1.33	1.11 - 1.58	0.0056*
3.Multiparous, excluding previous CS, single cephalic, ≥37 wks spontaneous labour	132	103	29	20.0	22.0	0.38	0.28 - 0.53	0.0000*
4.Multiparous, single cephalic, ≥37 wks induced labour or prelabour CS	35	20	15	5.3	42.9	0.85	0.57 - 1.25	0.3780
5.Previous CS, single cephalic,≥37 wks) (13.9%)	92	0	92	13.9	100.0	2.37	2.15-2.61	0.0000*
6.All nulliparous breeches)	19	0	19	2.9	100.0	2.05	1.89-2.22	0.0000*
7. All multiparous breeches (including previous CS)	10	0	10	1.5	100.0	2.02	1.87 - 2.18	0.0018*
8.All multiple pregnancies (including previous CS)	4	0	4	0.6	100.0	2.00	1.86 - 2.16	0.0631
9. All abnormal lies (including previous CS)	1	0	1	0.1	100.0	1.99	1.85 - 2.15	0.0000*
10.All single, cephalic, =<36 weeks (incl. previous CS)	58	43	15	8.8	25.9	0.49	0.32 - 0.77	0.0000*
Total	661	329	332	100	-	-	-	-

S7_Table 7_202501220329-1.tif This is a preview of your figure rendered on a simulated PLOS journal page.

Maecenas ac est sit amet odio sollicitudin euismod. In risus odio, convallis a neque ac, varius ultricies arcu. Vestibulum et quam iaculis, ultricies odio et, molestie magna. Suspendisse vehicula purus id turpis eleifend, et convallis dui dignissim. Praesent tempus elit a metus sollicitudin, sed fringilla nulla porttitor. Nullam in tempus massa. Nunc maximus magna massa, nec volutpat risus rhoncus ut. Fusce quis ante sem. Aenean nulla nibh, tempus sit amet rhoncus at, eleifend vel risus. Sed dictum, sem ultrices elementum pharetra, lacus diam volutpat orci, scelerisque semper dui lacus ut enim.

Suspendisse in nunc id lacus commodo consequat. Proin semper aliquam varius. Fusce vitae neque aliquam nisi ultrices sodales vitae ut enim. Vivamus nec dictum ipsum. Sed condimentum ante eu urna tincidunt tincidunt. In ac lacus nec ipsum viverra volutpat posuere vel lacus. Class aptent taciti sociosqu ad litora torquent per conubia nostra, per inceptos himenaeos. Morbi rhoncus ipsum quis lorem hendrerit, at vulputate massa tempus. Ut arcu nisl, gravida vitae risus ultricies, porta venenatis massa. Cras dignissim, enim at faucibus aliquam, sapien nisl eleifend dolor, vel mollis nulla nisi id ipsum. Pellentesque vehicula ultricies risus sit amet faucibus. Praesent sit
