# Supplementary material for: Explaining rising caesarean section rates in urban Nepal: A mixed-methods study
Source: PLoS One. 2025 Feb 26;20(2):e0318489. doi: 10.1371/journal.pone.0318489 (PMC11864527; doi:10.1371/journal.pone.0318489)
Supplement: S9 Table — (PDF) [file pone.0318489.s009.pdf]

Lorem Ipsum

Lorem ipsum dolor sit amet, consectetur adipiscing elit. Mauris maximus fringilla ligula, in malesuada erat tempor ac. Quisque dapibus posuere turpis, vel aliquam massa vehicula non.

Table 9: Association between mode of childbirth and sociodemographic/obstetrics characteristics

Sociodemographic/obstetrics Characteristics	Category	Mode of birth		P-value
		Normal (%)	C S (%)	
Types of hospital	PMWH	242(62.9)	143(37.1)	0.000*
	KMH	87(31.5)	189(68.5)	
Age of mother	<20	37(72.5)	14(27.5)	0.000*
	20-24	148(66.1)	76(33.9)	
	25-29	80(38.8)	126(61.2)	
	30-34	55(38.5)	88(61.5)	
	35+	9(24.3)	28(75.7)	
Gestational Age	<30	6(66.7)	3(33.3)	0.002*
	30-36	38(71.7)	15(28.3)	
	37-40	271(47.0)	306(53.0)	
	>40	14(63.6)	8(36.4)	
Parity	Nulliparous	200(52.6)	180(47.4)	0.130
	Multiparous	129(45.9)	152(54.1)	
Number of pregnancies	Single	329(50.1)	328(49.9)	0.135
	Multiple	0(0.0)	4(100.0)	
Foetal lie	Longitudinal	329(49.8)	331(50.2)	
	Transverse	0(0.0)	1(100.0)	1.000
Foetal presentation	Cephalic	329(55.2)	301(47.8)	
	Breech	0(0.0)	30(100.0)	
	Other	0(0.0)	1(100.0)	0.000*
Onset of labour	Spontaneous	267(65.4)	141(34.6)	
	Induced	62(81.6)	14(18.4)	
	No labour	0(0.0)	177(100.0)	0.000*
Birth other	1	200(52.6)	180(47.4)	
	2	92(46.0)	108(54.0)	
	3+	37(45.7)	44(54.3)	0.232
Apgar score at 1 minute	0-3	16(69.6)	7(30.4)	
	4-6	131(47.6)	144(52.4)	
	7-10	182(50.1)	181(49.9)	0.100
Apgar score at 5 minutes	0-3	14(77.8)	4(22.2)	
	4-6	8(53.3)	7(46.7)	
	7-10	307(48.9)	321(51.1)	0.050*
Birth weight	<2.5	65(50.4)	64(49.6)	
	2.5-3.9	261(50.1)	260(49.9)	
	>=4	3(27.3)	8(72.7)	0.300
Medical condition of mother	Yes	15(51.7)	14(48.3)	
	No	314(49.7)	318(50.3)	1.000
Bad/poor obstetric history	Yes	26(49.1)	27(50.9)	
	No	303(49.8)	305(50.2)	1.000
Sex of baby	Male	150(49.0)	156(51.0)	
	Female	179(50.4)	176(49.6)	0.800
Number of ANC visit	0	3(50.0)	3(50.0)	
	1-3	151(64.8)	82(35.2)	
	4+	175(41.5)	247(58.5)	0.000*
Religion	Hindu	230(48.8)	241(51.2)	
	Buddhist	85(53.8)	73(46.2)	
	Christian	10(40.0)	15(60.0)	
	Muslim	4(57.1)	3(42.9)	0.500
Ethnicity	High	109(42.2)	149(57.8)	
	Middle	207(55.1)	169(44.9)	
	Dalit	13(49.1)	14(51.9)	0.007*
Place of residence	Urban	152(44.1)	193(55.9)	0.003*
