# Supplementary material for: Explaining rising caesarean section rates in urban Nepal: A mixed-methods study
Source: PLoS One. 2025 Feb 26;20(2):e0318489. doi: 10.1371/journal.pone.0318489 (PMC11864527; doi:10.1371/journal.pone.0318489)
Supplement: S10 Table — (PDF) [file pone.0318489.s010.pdf]

PLOS

Lorem Ipsum

Lorem ipsum dolor sit amet, consectetur adipiscing elit. Mauris maximus fringilla ligula, in malesuada erat tempor ac. Quisque dapibus posuere turpis, vel aliquam massa vehicula non.

S10 Table: Bivariate/multivariate logistic regression selected variables by mode of childbirth

Variables	Category	OR	95% CI	P-value	AOR	95% CI	P-value
Types of Hospital	PMWH	Ref			Ref		
	KMH	1.37	1.27-1.47	0.0000*	1.32	1.22-1.44	0.0000*
Age of mother in years	<20	Ref					
	20-24	1.07	0.92-1.23	0.3800	1.05	0.93-1.17	0.4499
	25-29	1.40	1.21-1.62	0.0000*	1.17	1.04-1.32	0.0076*
	30-34	1.41	1.21-1.64	0.0000*	1.23	0.99-1.78	0.0652
	35+	1.62	1.32-1.98	0.0000*	1.29	1.09-1.51	0.0024*
Gestational age in week	<30	0.82	0.53-1.14	0.2374	1.25	0.90-1.74	0.1809
	30-36	0.78	0.68-0.89	0.0005*	0.94	0.85-1.05	0.2886
	37-40	Ref			Ref		
	≥40	0.85	0.69-1.05	0.1222	0.92	0.78-1.08	0.3158
Foetal presentation	Cephalic	Ref			Ref		
	Breech	1.69	1.45-2.02	0.0000*	1.08	0.93-1.25	0.3080
	Other	1.69	0.65-4.40	0.2900	1.00	0.48-2.12	0.9803
Onset of labour	Spontaneous	1.18	1.07-1.30	0.0012*	1.16	1.06-1.27	0.0016*
	Induced	Ref			Ref		
	No labour	2.26	2.03-2.52	0.0000*	1.96	1.76-2.18	0.0000*
Apgar score at 5 minutes	0-3	Ref					
	4-6	1.32	0.77-2.27	0.3140	1.39	0.99-1.93	0.0565
	7-10	1.33	1.06-1.69	0.0160*	1.39	0.98-1.99	0.0764
Number of ANC visit	0	1.16	0.78-1.72	0.4600	1.32	0.98-1.79	0.0732
	1-3	Ref					
	4+	1.26	1.17-1.37	0.0000*	1.26	1.06-1.19	0.0002*
Ethnicity of mother	High	1.14	1.05-1.23	0.0015*	1.00	0.95-1.07	0.8469
	Middle	Ref					
	Dalit	1.07	0.88-1.30	0.4861	1.04	0.90-1.21	0.5632
Place of Residence	Urban	1.09	1.01-1.17	0.0330*	0.98	0.92-1.04	0.4601
	Rural	Ref					

S10_table 10_202501221351-1.tif This is a preview of your figure rendered on a simulated PLOS journal page.

Maecenas ac est sit amet odio sollicitudin euismod. In risus odio, convallis a neque ac, varius ultricies arcu. Vestibulum et quam iaculis, ultricies odio et, molestie magna. Suspendisse vehicula purus id turpis eleifend, et convallis dui dignissim. Praesent tempus elit a metus sollicitudin, sed fringilla nulla porttitor. Nullam in tempus massa. Nunc maximus magna massa, nec volutpat risus rhoncus ut. Fusce quis ante sem. Aenean nulla nibh, tempus sit amet rhoncus at, eleifend vel risus. Sed dictum, sem ultrices elementum pharetra, lacus diam volutpat orci, scelerisque semper dui lacus ut enim.

Suspendisse in nunc id lacus commodo consequat. Proin semper aliquam varius. Fusce vitae neque aliquam nisi ultrices sodales vitae ut enim. Vivamus nec dictum ipsum. Sed condimentum ante eu urna tincidunt tincidunt. In ac lacus nec ipsum viverra volutpat posuere vel lacus. Class aptent taciti sociosqu ad litora torquent per conubia
