# Supplementary material for: Explaining rising caesarean section rates in urban Nepal: A mixed-methods study
Source: PLoS One. 2025 Feb 26;20(2):e0318489. doi: 10.1371/journal.pone.0318489 (PMC11864527; doi:10.1371/journal.pone.0318489)
Supplement: Attachment — Submitted filename: Review Comments.docx [file pone.0318489.s013.docx]

1. Please clarify the period and duration of the study in the abstract, similar to the manuscript, as the abstract has two different study periods (2021 & 2018-19).
2. Kindly provide the most recent CS rate report.
3. As the authors have mentioned 14 healthcare professionals, please ensure that you mention how many doctors, nurses, and midwives specifically.
4. Do you have any questionnaires based on which the interview was conducted?
5. Was the questionnaire validated? If so, mention the same in the methodology section of the manuscript. If the questionnaire was not validated, mention the same as limitations of the study.
6. Was pre-test done for questionnaire validation? If so, what is the percentage of the population on which the pretest was performed?
7. Please provide a table of demographic characteristics of the patients included in the study.
8. Ensure to provide figure legends for the figures.
9. Please mention the ethical approval number in the main manuscript.
10. Discuss the limitations of the study.
